# Organizational Strategy and Corporate Social Responsibility: The Mediating Effect of Triple Bottom Line

**DOI:** 10.3390/ijerph16224559

**Published:** 2019-11-18

**Authors:** Md. Abdul Kaium Masud, Md. Harun Ur Rashid, Tehmina Khan, Seong Mi Bae, Jong Dae Kim

**Affiliations:** 1 Department of Sustainability Management, Inha University, Incheon 22212, Korea; masud@inha.edu; 2 Department of Business Administration, Noakhali Science and Technology University, Noakhali 3814, Bangladesh; 3 Department of Economics & Banking, International Islamic University Chittagong, Chattogram 4318, Bangladesh; harunais88@gmail.com; 4 School of Accounting, RMIT University, Melbourne, Victoria 3000, Australia; tehmina.khan@rmit.edu.au; 5 College of Business Administration, Inha University, Incheon 22212, Korea; cyber2042@hanmail.net

**Keywords:** sustainability, corporate social responsibility, strategy, triple bottom line, mediation

## Abstract

Limited research in the area of the triple bottom line (TBL) mediation effect on the relationship between organizational strategic performance (OSP) and corporate social responsibility performance (CSRP) has motivated this study. The objective of this study is to investigate how OSP affects CSRP and the mediation impact of TBL elements through the decision-making process of business management. Considering a sample of 250 employees from Bangladesh, this study used structural equation modelling (SEM) to test the relevant research hypotheses. Through the lens of stakeholder, institutional, legitimacy and resource-based view theories along with rigorous statistical techniques, this study has found that OSP is positively related to CSRP. In terms of the mediation effect, this study has found that economic responsibility has no intervening role while environmental and social responsibility significantly mediated the relationship between OSP and CSRP. Finally, the full mediation power of the model suggests that OSP affects a firm’s strategic decision and CSR outcomes directly as well as indirectly through TBL.

## 1. Introduction

Corporate social responsibility (CSR) has been widely discussed in strategic management and organizational behavior literature due to it being a strategic priority for organizations—for example, for the purpose of maintaining legitimacy [1,2,3,4,5,6,7,8,9,10]. Strategic organizational planning is the broader use of strategic planning and analysis for the formulation of different policies and approaches such as strategic negotiations, stakeholder management, dynamic capabilities, contingency plans, rapid communications and greater awareness [8,10,11]. Prior strategic management literature has explained strategic planning as an important element of the innovative capability of management and a demonstration of the commitment to meet stakeholder expectations [10]. It has been considered as a trade-off between financial performance and innovative activity [12]. However, complexity arises with strategic ambiguity and around resource requirements for internal or external business development to attain strategic objectives [3,11,13]. Organizational strategic performance (OSP) is the execution of objectives set in the business to gain efficiencies to improve performance. Arend et al. [10] reported that flexible strategic planning has the potential to decrease financial performance while a reward-based, knowledge sharing and high risk-taking management approach enjoys maximum financial performance without trade-off [12].

Prior studies have explored the effectiveness, importance and performance of the organization regarding sustainability [1,9,14,15]. The sustainability concept captures different sustainable responsibility dimensions. Carroll [16] was the first to introduce four types of organizational responsibilities in the name of sustainability or CSR: economic, legal, ethical and discretionary responsibilities (see also Carroll [17]). Among the different concepts and dimensions, the triple bottom line (TBL) concept comprises economic, social, and environmental responsibility dimensions, and is the most prominent and recognized CSR or sustainability concept [9,18] (TBL is also considered as 3P; profit, planet and people).

Previous studies have documented the role of TBL for corporate social responsibility performance (CSRP) but most of the investigations were limited to the direct relationship (see [4,19,20,21,22,23,24,25]. On the other hand, OSP is a key concept in the development of TBL, while proactive OSP directly promotes TBL and CSRP [4,26,27,28]. Prior CSR, organizational behavior and strategic management studies have failed to recognize the intervening (mediator) influence of TBL between OSP and CSRP. For our purpose in this article, TBL covers organizational attractiveness [9,29,30], internal and external organizational performance [1,7,12], stakeholder satisfaction [31], corporate reputation [32], corporate visibility [15,21], transparency and accountability performance [33]. On the other hand, CSR is captured as impact. When firms perform TBL initiatives, they have an impact on CSR elements. Thus, we believe it could have a mediating or indirect impact on organizational strategic CSR actions and reporting.

This study aims to investigate the TBL mediation role between OSP and CSRP based on stakeholder and legitimacy theoretical underpinnings. To provide the empirical evidence, this study has used a survey questionnaire of 250 respondents from the manufacturing sector of Bangladesh. The reason for selecting the country is: (1) for the last couple of years, the country has extensively promoted CSR [19,34]; (2) the country is extremely vulnerable to global warming and climate risk [35], rendering CSR an instrument to mitigate these problems; (3) the manufacturing sector is considered less regulated and accountable [36]; and (4) no empirical research on the issue has so far been found to be undertaken.

## 2. Research Background and Theoretical Discussion

In strategic management literature, CSR has been treated as a strategic issue due to its dual nature of requiring resource allocation while generating strategic benefits such as enhanced reputation [2,6]. Chandler [37] argues that *“Yesterday’s ethical responsibilities can quickly become today’s economic and legal responsibilities. In order to achieve its fundamental economic obligations in today’s globalized world, a firm must incorporate a stakeholder perspective within its strategic outlook”* (2017, p. 6).

Prior study extensively used stakeholder theory [38], legitimacy theory [39], institutional theory [40,41], signaling theory [21] and resource-based view theory (RBV) [6,42] in the development and discussion of CSR and OSP. Most of the theories described many appropriate characteristics, determinants, and motivations for the relationship between CSR and OSP. Consistent with the studies by Zhang et al. [6], Federo and Saz-Carranza [8], Masud et al. [23], Colwell et al. [41], Madueño et al. [43], Garceia Sancez et al. [44], and Comyns [45], this study uses a mixed theoretical approach. Among them, stakeholder theory is mostly used to explain the diverse stakeholder needs, demands and how management responds to them through CSR. Stakeholder theory broadly explains the relationship between management and different stakeholders, including creditors, employees, suppliers, different communities, auditors, regulators, the media, non-government organizations (NGOs), investors, the government, customers, activist groups, national and international donor agencies, shareholders, and civil society [34,38]. Legitimacy theory defines the contract between the society and the organization, as the organization must follow social norms, values, and perceptions [46]. Organizations use CSR as a tool to communicate with and convince social stakeholders to eliminate different types of threats arising from business activities [45]. An organization’s strategic planning such as proactive and reactive strategies depends on the magnitude of the legitimacy between business and society [47]. On the other hand, institutional theory defines an organization as a field that must comply with different sets of internal and external rules, regulations and procedures [48]. Institutional theory defines the isomorphism process of an organization that is driven by coercive, mimetic and normative forces [45]. Doing business in the institutional environment, management must comply with the regulations and guidelines to create competitiveness and capability [41]. Further, organizational performance mostly depends on the top management commitment as strategic management must ensure explicit and implicit rules relevant to different organizational contexts [41].

Another important theoretical discussion broadly used in the relationship between strategic performance and CSR is resource-based view (RBV). According to RBV, organizational resources can be defined as *‘the tangible and intangible entities available to the firm that enable it to produce efficiently and/or effectively a market offering that has value for some market segment(s)’* [49] (1999 p. 128). Different types of resources such as financial, physical, technological, intellectual, legal, human, organizational, informational, or relational resources define organizational structures and their relationship with people, profit and planet. Organizational performance mainly depends on how these resources are effectively and efficiently used by the management. From the RBV perspective, organizational strategic factors that integrate, coordinate, and implement works into different projects, tasks and groups [42] are considered as resources. Prior research considered organizational strategic resources as having a unique and higher order that includes capital, technological, human, and policy-level resources [6]. Therefore, resource development is highly related to CSR performance as improvement or addition to the resources sends a strong signal to the market, society and stakeholders [21]. Based on the discussion, we believe that CSR performance and organization strategic improvement is mostly defined by the institutional environment while management is motivated to use proper resources to create legitimacy among institutions, stakeholders and society.

## 3. Literature Review and Hypothesis Development

### 3.1. Organizational Strategic Performance and Corporate Social Responsibility Performance

Prior literature has documented the relationship among OSP, organizational financial performance and CSRP [3,4,9,10,12,15]. Most of the studies have found that OSP acts as a crucial consideration for undertaking sustainable development initiatives and is implemented as growth and market competitiveness rather than traditional profit maximization [8,10,11,13,50]. It is also evident that OSP is the key determinant of CSRP because strategic planning is a comprehensive consideration and details an outline of the firm’s overall performance (e.g., industry, sector, market, competitors, technologies, and growth). Prior literature also explored the success or failure of the organization being impacted by the effectiveness and efficiency of strategic planning and the execution of the plan. According to Bryson, OSP is *‘a deliberative, disciplined effort to produce fundamental decisions and actions that shape and guide what an organization is, what it does, and why it does it’* [51] (2011, pp. 7–8). Recardo [3] distinguishes between capital “S” and lowercase “s” to define organizational strategy where the big “S” defines a firm’s long-term internal and external planning (e.g., market, joint ventures, mergers and acquisition, and collaboration) and the small “s” is about market forces (e.g., price, place, and promotion). Strategic planning is the decision of the senior or the top-level management that is represented in the corporate vision, mission, goals and target strategy [8,15]. Therefore, in an organizational strategic system, planning and motion should be aligned with triple bottom line approaches to gain maximum benefits of internal competencies combined with external opportunities. Additionally, OSP enhances a sustainability vision that encourages organizations’ economic, environment and social responsibility [15].

To gain competitive advantages, organizational strategy and strategic planning must be acceptable to a wide range of stakeholders (e.g., customers, employees, shareholders, suppliers, creditors, government, media, competitors, local authorities, and international alliances). CSRP depends on the organizational reactive or proactive strategy. Reactive strategy undermines CSR because of limited stakeholder engagement and management incapability whereas proactive strategy searches for the future responsibilities beyond the social standards and promotes CSR [2,52]. Therefore, sustainable strategic proactive planning and implementation facilitate organizational policies and procedures in order to provide, develop, control and monitor economic, environmental and social responsibilities. Kanji and Chopra [4] reported that OSP positively influences CSR to achieve organizational accountability and benefits. Arend et al. [10] stated that superior strategic planning positively affects organizational financial performance that directly and indirectly promotes risk-taking and knowledge-based management. Shaukat et al. [28] and Clarkson et al. [47] also argued that organizational proactive strategy positively and significantly promotes social and environmental performance. Moreover, Marin et al. [2] showed that an innovative strategy significantly influences competitiveness that is directly related to CSRP, whereas Arend et al. [10] reported that strategic performance negatively affects innovation in firms. Based on the above discussion, the following hypothesis has been developed:

**Hypothesis** **1** **(H1):** *OSP has a positive relationship with CSRP.*

### 3.2. The Mediating Role

Generally, mediator variable is a third variable that explains how or why two other variables are related. Therefore, the mediation model is a three-variable model where an independent variable (X) predicts a mediator variable (M) which in turn determines an outcome (Y) [53,54]. The mediator intervenes to explain the process by which the two variables, X and Y, are related. The mediating variable could partially or fully mediate the relationship between X and Y [53]. In prior research of CSR and OSP, the mediation effect was tested frequently to determine the complex mechanism that relates dependent with independent variables [1,7,41,43].

#### 3.2.1. The Mediating Role of the Organizational Economic Responsibility

Many studies explored the relationship between OSP and TBL in the broad range of organizational CSRP. Corporation performance depends on the strategic governance and leadership that is directed by sound policy and execution. Strong market competition, rapid technological changes, changing buying behavior and many other factors directly and indirectly influence organizational decision-making processes as well as CSR. According to the stakeholder theory, organization must consider stakeholder engagement to enhance financial as well as non-financial performance [38]. Stakeholder activism regarding sustainable issues is more concerning as stakeholders raise many questions and queries regarding financial decision making, ethical matters, sound governance, and corporate accountability. Moreover, strong national and international non-government organizations (NGOs), traditional and social media, and local authority often create pressures on organizations regarding compliance and undertaking CSR activities. Therefore, proactive strategic management considers present and future market forces in both the short- and long-term strategic planning and policy formulation and execution that ultimately enhance CSRP.

To overcome stakeholder pressures, management considers several functions that promote legitimacy with society and stakeholders. In line with TBL, economic responsibility directly promotes four types of economic benefits for stakeholders [4]. Kanji and Chopra [4] identified profitability, transparency, non-discrimination and investing in sustainability as crucial economic responsibilities of organizations towards investors, employees and customers. Prior literature also bears evidence that OSP has a direct influence on economic responsibility as well as on CSRP. On the other hand, we like to believe economic responsibility is an influential element of TBL that directly and indirectly affects CSRP. Economic responsibility under TBL should promote strategic planning regarding the allocation of resources for CSR. The following hypothesis is proposed.

**Hypothesis** **2** **(H2):** *The relationship between OSP and CSRP is mediated by organizational economic responsibility performance.*

#### 3.2.2. The Mediating Role of the Organizational Environmental Responsibility

Sustainable vision and mission mainly focus on environmental sustainability performance as business management is accountable and responsible to the natural environment (Savitz, 2014). Since the environment is the root of sustainability, strategic planning regarding corporate environmental policy, carbon risk and reduction, environmental management systems, business strategies for climate change, the reduction of air and water pollution, biodiversity policy, waste and recycling management and corporate environmental disclosure have a significant impact on CSRP [19,25,55,56,57].

In order to mitigate global warming and climate risks, business management must ensure technological advancement, research and development initiatives and sound environmental management practices. A sound proactive environmental strategy promotes risk management, responsibilities and accountabilities of the corporation that will enhance competitive advantages and market growth [26]. Managing environmental responsibility investments, green human resource management and internal environmental performance sends a strong signal to the diverse stakeholders, promotes reputation and mitigates different activist group concerns regarding business impacts and relevant environmental issues [21]. Moreover, the strategic environmental policy reduces possible business risks and gains potential benefits. For example, the CEO of Cola-Cola incorporated a vision 2020 in the year 2010 and declared to reduce CO_2_ emission by 25% and they reduced CO_2_ emissions by 19% by the end of 2017. A prior study also found that OSP deliberately influences CSRP because of its active role in the TBL [4,9]. Effective and sound strategic planning mitigates possible risks and threats from the destructive business policy and helps to pacify different stakeholders by implementing a responsible environmental strategy. Most of the studies have focused on the role of OSP and CSRP but have not addressed the measurement of the individual role of environmental responsibility under TBL. This study attempts to investigate the mediating role of environmental performance between OSP and CSRP. Therefore, we develop the following hypothesis:

**Hypothesis** **3** **(H3):** *The relationship between OSP and CSRP is mediated by organizational environmental responsibility performance.*

#### 3.2.3. The Mediating Role of the Organizational Social Responsibility

**Figure 1:**
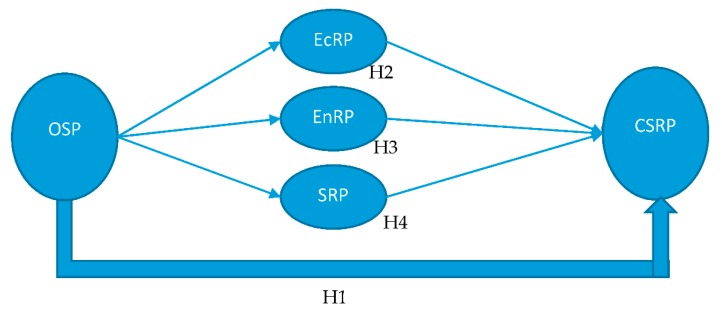
Conceptual framework.

According to the stakeholder theory, organizations face numerous internal and external pressures for being a sustainable corporation. Mitigating internal pressures (ensuring good working environment, standard wages, equal employment opportunity, trade union facility) and undertaking external responsibility (social compliance, anti-corruptive behavior, child labor policy, employment opportunity, and product responsibility) are critical elements of strategic planning and policy. To be a socially responsible corporation, management needs to undertake relevant changes and updates in policies and practices to improve social and environmental performance. For example, in the year of 2010, Coca-Cola introduced the vision 2020 strategy to mitigate many social, environmental and economic problems (e.g., obesity and health, water, supply chain, and women empowerment). Based on social legitimacy theory, business management must take care of social responsibility to gain long-term benefits and social acceptance [39]. Therefore, proactive organizational strategic planning and policy ultimately improves CSRP through TBL approaches. For instance, Unilever reshaped CSR strategy as *sustainable living plan* in the year 2010 to be a global sustainable leader and has become the corporate leader in sustainability [37,58]. Prior literature has also found that OSP has a significant impact on social performance, whereas social performance directly promotes CSRP as an essential part of TBL [4,19]. Moreover, social responsibility is the ultimate action of OSP, while potential benefits are driven from the execution of CSRP. We found that no prior study investigated the mediating role of social responsibility between OSP and CSRP. Therefore, to find the mediating role, we propose the following hypothesis:

**Hypothesis** **4** **(H4):** *The relationship between OSP and CSRP is mediated by organizational social responsibility performance (Figure 1).*

## 4. Research Methodology

### 4.1. Data Collection and Sample

The empirical study has used primary sources of data collected by the questionnaire survey. Three hundred (300) questionnaires were distributed based on random stratified sampling among the employees of various manufacturing industries in Bangladesh during the period of July 2017 to December 2017. In the middle of 2018, a sample of 255 responses was returned, showing a high response rate (85%) that is consistent with Madueno et al. [43], Ramesh et al. [1] and Hur et al. [7]. As stated earlier, this study is based on the manufacturing sector since manufacturing firms are the major listed firms in the stock exchange of the country (i.e., 40% of firms are from the manufacturing sector). Moreover, manufacturing firms are considered more responsible for global warming and climate change and, therefore, CSR activities and functions are more likely supported by the manufacturing industries [36,59]. Our choice of manufacturing industry sample is consistent with recent CSR studies by Muttakin et al. [36] and Khan et al. [60]. Five multivariate data (responses) were detected and filtered out from the surveyed data during the test of Mahalanobis distance (D2) using the critical Chi-square function at ρ<0.01. There were no univariate issues in this research. The final sample consists of 250 responses, indicating a high response rate from the survey (83%) (see Table 1), compared to the recent study by Ramesh et al. [1], Hur et al. [7], Bohlmann et al. [9] and Gao et al. [14]. Having three or more indicators per factor, a sample size of 150 will usually be sufficient for a convergent and proper solution-based research study [61].

### 4.2. Measurement Items and Instruments

This study used Brislin’s [62] back-translation procedure. The original questionnaires were translated into Bangla and again translated into English for data analysis purposes. The back-translated questionnaire was further checked by several management scholars to ensure the Bangla and English version were equivalent to each other and acceptable.

This study used a 7-point Likert scale, a closed-ended structured questionnaire to measure OSP, Economic Responsibility Performance (EcRP), Environmental Responsibility Performance (EnRP), Social Responsibility Performance (SRP), and CSRP, where one indicates strongly disagree and seven indicates strongly agree. The questionnaire was mainly developed based on the prior study by Kanji and Chopra, [4] (also known as the Kanji and Chopra CSR model). The five latent variables address the operationalization of the manufacturing firms in relation to strategic policy, competitive advantages, and commitment to green and environmental performance, management responsibilities towards employees, community, society and customers. The questions also evaluate how the employees value their organizations. Moreover, the questionnaire was split into two segments: demographic and the item related to observed variables.

The demographic data includes the frequency and percentage of types of manufacturing industries, the number of employees in the industry, employees’ status and number of years in operation (Table 1). Among the five industries, with the highest percentage of respondents, there were 112 (49%) automotive respondents. The lowest percentage (1%) of respondents were from the glass industry. Most of the respondents were from large-scale manufacturing (74%) firms in the country. In this study, mid-level management participation (61%) was higher than first-line level management (37%). Moreover, 81% of the respondents were highly experienced.

In addition to the demographic information, the questionnaire consists of a total of thirty-one (31) items, with five (5) latent variables that are CSRP (7), OSP (5), organizational EnRP (6), organizational EcRP (5) and organizational SRP (8). Our questionnaire design to construct the five latent variables is validated by all statistical inferences consistent with prior studies [1,7,41,43]. We asked employees of the manufacturing firms how they recognize and support management planning, consistent with the study by Madueño et al. [43] (see also [63,64]). Our latent variable EnRP, EcRP and SRP were designed to identify employees’ perceptions of TBL responsibility, accountability and performance according to Madueño et al. [43]. Our latent variable OSP was considered to trace management capacity building, innovative attitudes, and competitive performance according to prior studies [43,65]. The detailed explanation of five latent variables is given in Table 2.

It is also notable that in previous behavioral research, a minimum of one to a maximum of ten questions were constructed to ascertain employees’ perceptions of top management [1,7,41,43,66]. For example, Madueño et al. [43] asked nine questions on environmental performance and four questions on the customer perception of CSR responsibility of Spanish firms. Colwell et al. [41] used a maximum of ten questions and a minimum of four questions to develop several latent variables in the study to determine top management responsiveness regarding organizational performance. Moreover, Chenhall [65] developed different organizational strategic latent variables by asking a minimum of three to a maximum of five questions, while latent variables were constructed by a different set of questionnaires (i.e., in strategic and operational linkages, latent variables were created by twelve questions and these questions were subdivided into three groups).

As stated earlier, this study considered latent variables based on TBL approaches. This study applied confirmatory factor analysis (CFA) to generate the latent variables. Cronbach’s alpha coefficient (CA), average variance extracted (AVE), and composite reliability (CR) were run to test the internal consistency and reliability of the data. Finally, structural equation modeling (SEM) was conducted for hypothesis testing. Statistical packages SPSS 22 and Smart PLS 3 were used to perform the analysis.

## 5. Results and Analysis

### 5.1. Descriptive Statistics and Correlation Matrix

Table 3 presents the means, standard deviation (SD) and the correlation between each variable in the diagonal elements. The results show a very strong correlation among the variables (*p* < 0.01). CSRP is positively and significantly correlated with OSP, EcRP, EnRP and SRP. No correlation value exceeds the critical value of 0.90, indicating no multicollinearity problem [67]. Additionally, the highest value of Pearson correlation between OSP and SRP is 0.649, which is less than the recommended value of 0.90, alleviating the multicollinearity concern of the five variables [67].

### 5.2. Internal Consistency, Reliability and Validity Testing

This study applied the KMO and Bartlett’s test to check the sample size. The value of KMO is 0.926, which is greater than the threshold of 0.77 [68]. Therefore, the sample size is adequate for factor analysis. The result of Bartlett’s test (6668.393, df = 741, ρ<0.01) explains that the correlation is meaningfully different from the identity matrix and the correlations among the factors are not zero. Table 4 shows the internal reliability and convergent validity measures of this study. The results of CA of all variables ranged from 0.860 to 0.934, which is greater than the threshold 0.77 [69]. Moreover, CR and AVE were used to measure the internal consistency among the variables. The value of CR and AVE are also higher than the limit of 0.70 and 0.50 respectively as recommended by Bagozzi and Yi [70], which reveals that all variables are internally consistent and reliable.

Additionally, discriminant validity of the model is assessed in Table 5. Indicators should load more strongly on their own constructs than on other constructs in the model, and the average variance shared between each construct and its measurements should be greater than the variance shared between the construct and other constructs [71]. The result indicates that all constructs exhibit sufficient or satisfactory discriminant validity [71], where the square root of AVE (diagonal) is larger than the correlations (off-diagonal) for all reflective constructs

Table 6 presents the factor loadings of the five latent variables. This study finds the factor loading values of all items above 0.665, which is greater than the threshold 0.55 according to Tabachnick and Fidell, [72] and Hur et al. [7]. All factor loadings greater than the threshold confirm that the rotated component matrix consists of the final thirty-one items. In addition, the values of communalities are also over 0.5, which indicates the extent to which an item correlates with all other items. However, these five latent variables of this study successfully explain 65.69 percent of the impact, and environmental responsibility is the most pronounced (35.61 percent) in explaining the variance. It is noteworthy that economic responsibility variance has the least impact among the five latent variables (5.07 percent).

### 5.3. Confirmatory Factor Analysis

The efficiency of the CFA model highly depends on the measurement of the fitness of the model. The present study applied CFA at two stages following Ramesh et al. [1] and Hur et al. [7]. Initially, CFA was conducted in a one-factor model, considering the CSRP as a dependent variable and OSP as an independent variable, and then three mediating variables (EcRP, EnRP and SRP) were added in the mediation model. In both stages, this study found a good fit index (Table 7). For the one factor-model, the result showed (χ2(53)=100.324; ρ<0.05, CFI=0.976, TLI=0.970, SRMR=0.25 and RMSEA =0.06), whereas, in the final mediation model, the goodness of fit index demonstrated (χ2(427)=804.383; ρ<0.05, CFI=0.924, TLI=0.917, SRMR=0.52 and RMSEA=0.06), which are all above the threshold level [1,7,73,74]. Thus, the overall model fitness verified the superiority of our hypothesized model. In addition, Table 6 also shows that the proposed model is a good fit, with 31.5 percent, 26.4 percent and 54.6 percent of the total variance explained by EcRP, EnRP and SRP, respectively. Furthermore, this study found that the mediating model explanation power of CSRP (54.6 percent) is higher than the initial stage model CSRP (24.6 percent).

### 5.4. Hypothesis Testing

The results of structural equation modeling (SEM) are presented with the path coefficient, standard error (SE), critical ratios (CR) and *T*-statistics of the direct effects in Table 8. This study itemized and examined a multiple sequential mediation model [75].

**Figure 2:**
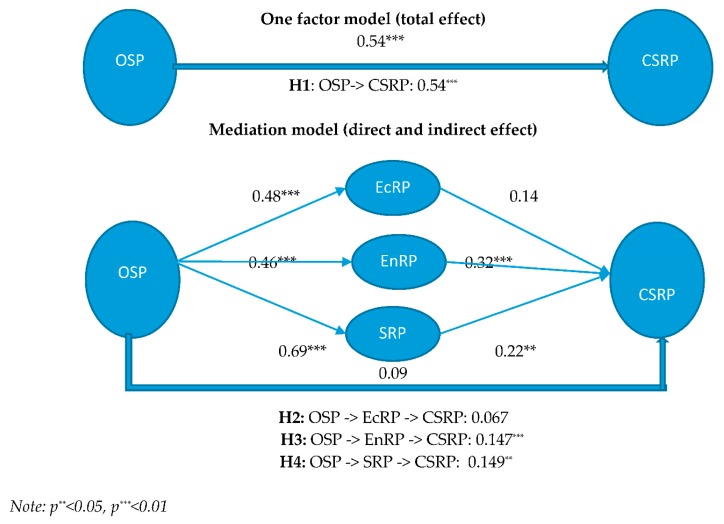
Research model and hypothesis testing.

Figure 2 represents the path coefficients and hypothesis test results. This study followed the three-step process of Hur et al. [7] to test the four hypotheses. This study hypothesized that the relationship between OSP and CSRP is partially or completely mediated by the TBL factors of EcRP, EnRP and SRP. At the first step, we posited that OSP was positively related to the CSRP and the results supported H1 (β=0.054, ρ<0.01), as shown in the upper part of Figure 2, which is consistent with the previous studies [9,10]. Second, six paths were investigated to examine the three mediation effects of the model presented in the lower part of Figure 2. We confirmed the significant direct relationship between OSP and SRP (β=0.686, ρ<0.01); OSP and EnRP (β=0.458, ρ<0.01); and OSP and EcRP (β=0.481, ρ<0.05). Similarly, we also found the significant relationship between SRP and CSRP (β=0.217, ρ<0.01), and EnRP and CSRP (β=0.321, ρ<0.01). However, the relationship between EcRP and CSRP turned out to be insignificant (β=0.138, ρ>0.10), suggesting that EcRP has no influence on CSRP. Third, we estimated these indirect effects by the bootstrap analysis. Our first bootstrapping mediating variable OSP -> EcRP -> CSR, (β=0.067, t=1.523) was found to be statistically insignificant, leading to the rejection of H2. The bootstrapping analysis of the remaining mediating variables shows the two significant indirect effects: OSP -> EnRP -> CSR (β=0.147, t=4.274, ρ<0.01) and OSP -> SRP -> CSR (β=0.149, t=2.550, ρ<0.05), supporting H3 and H4. In sum, this study found a strong and significant direct relationship between OSP and CSRP before introducing the mediation effects of TBL factors. The direct relationship between OSP and CSRP becomes insignificant with the three mediation variables included, which supports a complete (full) mediation of the model.

## 6. Discussion

Organizational strategic planning, analysis, and execution have been considered as the most tactical part of doing business and gaining organizational as well as financial performance. CSR has been considered as an organizational responsibility to the different stakeholders of society. CSRP ensures corporation willingness, responsibility and accountability towards diverse sets of stakeholders as it is considered a strategic tool for a corporation. Strategic CSR broadly lies in the TBL approaches of economic, environmental and social responsibility that is determined by the business management’s proactive or reactive strategy. Therefore, better CSRP is the ultimate result of a corporation’s OSP and TBL, where TBL plays an important role in relation to competitive advantage, reputation and capability. Prior literature exponentially documented the role of OSP in corporate CSRP along with its direct influence on TBL approaches, but not a single study examined the mediating role of TBL approaches in the OSP and CSRP relationship. This is the first study to investigate the mediating role of economic, environment and social responsibility performance of the manufacturing industry of Bangladesh. This study explored the TBL mediating role in the OSP and CSRP relationship by data analysis of a questionnaire survey of 250 employees during the period 2017–2018. Using the mixed theoretical lens of stakeholders, social legitimacy, institutional and resource-based view theories, this study reports that TBL has a strong mediating role between OSP and CSRP. This study showed that effective implementation of OSP improves environmental and social responsibility performance, which, in turn, enhances CSRP. On the other hand, we argue that without proper environmental and social performance, OSP will be unable to promote an organization’s internal and external risk management and to take advantage of opportunities for competitive advantage.

In this study, we found that OSP is positively and significantly associated with CSRP without TBL mediating effects. However, the direct relationship becomes insignificant with the TBL mediating role although the relationship remains positive. Our result is consistent with prior studies [28] (Shaukat et al. 2016 [26], Dou et al. 2017 [12], Song et al. 2011, and [19] Sultana et al. 2018) with the argument that proactive and sound strategic management significantly promotes CSRP. The results argue that proactive strategic planning is inevitable for better CSRP. Moreover, sound CSRP denotes effective stakeholder management, and sustainable strategic planning to promote societal legitimacy. Additionally, the direct relationship posits that business management of the manufacturing firms in Bangladesh effectively incorporates and manages organizational strategic planning to promote CSRP. The empirical finding is also consistent with the recent studies of Sultana et al. [19], Masud et al. [22], Muttakin et al. [36], Masud et al. [56], and Hoque et al. [57], and while these empirical studies argued that Bangladeshi firms are seriously considering environmental, social and economic responsibility in corporate decision making and action. Moreover, Masud et al. [34], Masud et al. [76], and Bose et al. [77] have posited that local regulation (CSR rules, green finance guidelines, money laundering rules, and environmental risk assessment guidelines) as well as international CSR guidelines (e.g., GRI-Global Reporting Initiative, ISO- International Organization for Standardization) have had a strong impact on Bangladeshi firms to promote sustainable management practices.

We found that OSP directly influences EcRP, while EcRP has no influence on CSRP. The result has shown that EcRP has no room to intervene between OSP and CSRP. It confirms that EcRP is not an intervening (mediator) variable. We explain this finding by arguing that profitability or revenue generation is the ultimate goal of the firms, and, thus, the shareholder profit maximization or wealth maximization concept particularly promotes profitability rather than sustainable economic elements. Additionally, TBL economic factors are more concerned about broader economic development and growth. Philanthropy, sponsoring community projects, spending on social betterment are all elements of TBL economic performance and as such it varies between countries as it depends on specific social and economic systems [78]. Our result is consistent with a previous TBL disclosure study in which no direct relationship between profitability and economic responsibility disclosure was found [55]. Moreover, Bae et al. [21] provided evidence that in relation to sustainability management disclosure, economic disclosure is the least prominent to demonstrate organizations’ corporate governance and strategic mechanisms.

We have investigated the mediating role of EnRP between OSP and CSRP. We found that both OSP and CSRP are highly mediated by the EnRP. This study revealed that EnRP has the power to mediate the relationship between OSP and CSRP. The results showed that sound environmental performance is the key element to be a sustainable corporation, as mitigating global warming, climate risks, carbon risks, biodiversity loss, air and water pollution, addressing waste and hazardous problems are required to implement a comprehensive environmental policy. Our result is also in line with stakeholder and legitimacy theories, as these theories support the concept of stakeholder concerns for an organization’s environmental initiatives such as technological investment, green investment, and environmental management systems [19,79,80]. Moreover, environmental performance reduces societal as well as political pressure and promotes legitimacy in society. Additionally, environmental performance conveys a prominent signal to the market that helps business management enhance competitive advantages [21].

In the last stage, we tested the mediating role of SRP with OSP and CSRP. We documented significant effects between OSP and SRP and between SRP and CSRP. We also found that SRP has a mediating power between OSP and CSRP. Organizations’ overall performance is highly dependent on the effective management of different social and organizational elements such as workplace health and safety conditions for employees, sound labor practices such as fair wage, accountability and transparency, anti-corruptive behavior, sound human resource policies, and women empowerment policies. SRP enhances internal stakeholder privileges (for corporations’ employees, workers, and different level managers) as well as external parties’ satisfaction and opportunities (from the perspective of investors, civil society, media, local authorities, national and international non-profit organizations and activist groups). We showed evidence that a sound OSP will be not enough to achieve CSRP unless a strong SRP is considered in the decision-making process. Finally, we found that TBL elements have complete (full) mediating effects between OSP and CSRP. Our study contributes to the organizational behavior and business ethics literature by investigating business strategic management in the context of TBL. Our empirical findings suggest that to be responsible and ethical, a firm’s management must consider TBL elements, especially environment and society, in the strategic decision process.

## 7. Conclusions and Contributions

Our research findings have substantial implications for business management, especially in the policy and strategy area. In both stages (before mediation and after mediation), OSP has a positive relationship to CSRP, which suggests that organizational strategic planning, analyzing, and execution are very influential in achieving maximum CSRP. Moreover, the complete mediation role of TBL denotes that business management and policy makers must show commitment to TBL performance. For example, if a corporation has sound climate risk policy and has already declared the target to reduce CO_2_ but the implementation of the CO_2_ reduction is poor, this low environmental performance will definitely have a negative impact on CSRP, and the organization will receive huge pressure from the different environmental groups, civil society, media and the government. This study finds that poor outcome in TBL elements have a potential to threaten societal and stakeholder relationships and it will widen the legitimacy gap. Moreover, irresponsible performance from the TBL approaches will drastically compromise the organizations’ competitive benefits, reputation and market growth. The empirical findings will also help management to provide more attention to environmental and social responsibility performance, as these two elements significantly mediate between OSP and CSRP. Additionally, this study contributes to determining the importance of policy making and implementation. For instance, effective labor and human resource policy, anti-corruption and whistleblowing policies indicate organization commitment and strategy against discrimination and corruption. However, if there is a strong allegation of the organization being involved in corruption, bribery, unethical and informal transactions, it will suggest ineffective and poor TBL performance.

Our empirical finding brings new insights into the CSR and TBL research domains by discovering the TBL mediating role for organizational strategic and corporate social responsibility performance enhancement. Although, a good number of studies have examined the direct relationships between organizational strategic issues and CSR outcomes, our study is the first to demonstrate the mediating role of the three important elements of CSR or sustainability concept based on various theories. Theoretically, this study emphasizes that the TBL approach has full intervening power. Additionally, this study contributes to stakeholders and legitimacy theoretical perspectives by highlighting that environmental and social responsibility elements are the most influential and essential factors to determine the relationship between organizational strategy and corporate responsibility. Furthermore, from the RBV theoretical perspective, this study emphasizes that an important element of TBL, the economic responsibility factor, must be reshaped to hold firms accountable not only with their environmental but also social and economic impacts. Firms need to think beyond profit maximization, towards implementing measures for improving community welfare.

This study also highlights that organizational strategic performance as well as CSR responsibility are the mechanisms of sound resource mobilization, capacity development, and proper utilization of the diverse resources. Additionally, our empirical study also has a significant contribution to the institutional environmental setting in response to TBL responsibility. Coercive, mimetic, and normative pressures substantially influence top management to comply with the regulations, guidelines and business responsibilities that ultimately enhance CSR performance. Finally, our study contributes to the organizational behavior and business ethics literature by investigating responsible symbiosis between business management and employees in the context of TBL. Our empirical findings suggest that a firm’s management must consider TBL elements in the strategic decision process to be responsible and ethical.

The contributions of this study are limited by several factors. First, this study is based on the manufacturing sector. Second, the survey of this study only considered employees, while other stakeholders were not approached. Third, this study was conducted in Bangladesh, a developing country; the outcome may not be applicable to developed country contexts. Finally, this study has a limitation in that the questions measuring some latent variables have conceptual similarities (e.g., CSRP and SRP). CSRP is the accumulation of an organization’s different functions and activities that might be the results of TBL performance. As we stated earlier, the questionnaire was taken from prior studies but the limitation has to be overcome in future studies. In future studies, the questions should be redesigned to ensure distinct constructs between TBL and CSR performance. Mixed research methodologies can be applied in future studies to best understand the psychological and ethical attitudes of business management in the context of CSR and triple bottom line performance.

## Figures and Tables

**Table 1:** Sample distribution.

Items	Categories	Respondents	Percentage (%)
Industry	Steel	72	28.8
	Glass	2	0.8
	Textile	64	25.6
	Automotive	112	44.8
Employees	Below 20	14	5.6
	20–50	52	20.8
	Over 100	184	73.6
Management	Top level	4	1.6
	Middle level	153	61.2
	Lower level	93	37.2
Operation	Less than ten years	18	7.2
	10–20 years	29	11.6
	Over 20 years	203	81.2

**Table 2:** Variable definitions.

Variables	Definitions
Organizational Strategic Planning (OSP)	OSP is the broader use of strategic planning and analysis for the formulation of different policies and approaches such as strategic negotiations, stakeholder management, dynamic capabilities, contingency plans, rapid communications and greater awareness. Organizational internal and external performance broadly cover sound strategic planning. For example, Unilever’s sustainable living plan 2020, Coca-Cola’s CSR strategy 2020, General Motor’s environmental strategic plan and Patagonia’s green strategy effectively helped them to be global leaders in sustainability. Therefore, OSP leads to achieving the sustainability target of the firm. The questions are designed to measure the management initiatives regarding organizational short- and long-term sustainability management.
Environmental Responsibility Performance (EnRP)	EnRP represents the ability of manufacturing firms to reduce environmental impacts through the reduction of carbon emissions, waste management, and automatic product design to control the risk of environmental hazards and the effect of climate change. In sustainability, field environmental sustainability is the top priority of the management. Environmental responsibility and performance increase productivity as well as market visibility of the manufacturing firms to diverse stakeholders. This study considers questions that explain management initiatives to control the risk of environmental accidents, reduction of physical hazards and technological improvement in the production process that allow ensuring environmental performance.
Economic Responsibility Performance (EcRP)	EcRP explains a firm’s ability to maximize profitability by reducing costs as well as improving the quality of life of employees. Economic performance encourages corporate management to take sustainable initiatives. This study considers the specific questions of EcRP in the ground of various stakeholder protections and opportunities, quality life of the workforce, employment opportunities and economic development of the country. All questions are designed to explain the employees’ perception of the corporation’s economic sustainability progress.
Social Responsibility Performance (SRP)	SRP represents organizational performance regarding social legitimacy that ensures strong bonding between business and society. Organizations are doing business in society and, as a result, they have a huge responsibility to care for the social stakeholders. Therefore, the organization has to undertake good initiatives to ensure a healthy workplace, standard wage policy, safety and security and freedom of expression of the workers. Ensuring opportunities and benefits to the internal workers, SRP broadly acknowledges the responsibility to the whole society. For example, Unilever set a target that, by 2020, they would help over a billion people improve their health and well-being. Moreover, Hindustan Unilever achieved success in empowering rural women by job creation projects that also increased revenues. This study considers questions about SRP in line with internal employees’ benefits, opportunities, and access to power that represents the organization’s perception of the society. The questions broadly define the ethical responsibly of the corporation as well as accountability to the diverse stakeholders.
Corporate Social Responsibility Performance (CSRP)	CSRP states the contribution of the organization to the different stakeholders of the society including employees, customers, investors, community people, government, local authority, media, civil society, and environmental activists. CSRP creates values for the organization, both internally and externally. Kanji and Chopra [4] define CSR as “an integral component of the operations of a company whereby it voluntarily contributes to society in terms of economic, environmental, ethical and social investment.” Organizational strategic planning and short- and long-term policy and strategy as well as CSR activities are designed to enhance CSRP. CSRP is an important tool that allows the organization to overcome all possible drawbacks by considering appropriate CSR programs and functions. This study considers the CSRP questions based on the broad sense of the organization that covers both internal and external CSR philosophies of the corporation. The CSRP questions explicitly and implicitly denote ethical responsibility, accountability, awareness, and transparency of the organization. Moreover, CSRP also alludes to determining the corporate management’s willingness in terms of the implementation of TBL approaches.

**Table 3:** Means, standard deviations, and Pearson correlation.

Variables	Mean	SD	OSP	EnRP	CSRP	SRP	EcRP ^1^
OSP	6.28	0.64	1				
EnRP	5.94	0.66	0.459 **	1			
CSRP	6.10	0.62	0.456 **	0.532 **	1		
SRP	6.16	0.53	0.649 **	0.462 **	0.508 **	1	
EcRP	6.02	0.70	0.477 **	0.548 **	0.465 **	0.531 **	1

** Correlation is significant at the 0.01 level (two tailed). ^1^ OSP = organizational strategic performance; EnRP = Environmental responsibility performance; SRP = Social responsibility performance; and EcRP = Economic responsibility performance.

**Table 4:** Reliability and convergent validity check.

Variables	Cronbach’s Alpha Coefficient (CA)	rho_A	Composite Reliability (CR)	Average Variance Extracted (AVE)
OSP	0.934	0.935	0.950	0.791
EnRP	0.928	0.930	0.943	0.735
CSRP	0.895	0.902	0.917	0.613
SRP	0.873	0.877	0.901	0.533
EcRP	0.860	0.862	0.900	0.642

**Table 5:** Discriminant validity.

Indicators	CSRP	Economic	Environment	OSP	Social
**CSRP**	**0.783**				
**Economic**	0.474	**0.801**			
**Environment**	0.539	0.550	**0.857**		
**OSP**	0.454	0.481	0.458	**0.889**	
**Social**	0.500	0.529	0.458	0.686	**0.730**

**Table 6:** Factor loading.

Indices	Items	Loading Factors	Communalities	Eigen Value	Variance (%)	Cum (%)
Environmental responsibility performance	EnRP20	0.827	0.773	12.186	39.309	39.309
	EnRP24	0.801	0.782			
	EnRP23	0.794	0.774			
	EnRP22	0.793	0.724			
	EnRP21	0.790	0.770			
	EnRP19	0.679	0.629			
Corporate social responsibility performance	CSRP28	0.767	0.635	2.780	8.969	48.278
	CSRP30	0.736	0.611			
	CSRP31	0.729	0.670			
	CSRP29	0.723	0.620			
	CSRP26	0.705	0.624			
	CSRP25	0.701	0.657			
	CSRP27	0.665	0.592			
Organizational strategic performance	OSP1	0.825	0.814	2.079	6.706	54.984
	OSP4	0.805	0.796			
	OSP2	0.778	0.802			
	OSO5	0.756	0.716			
	OSP3	0.748	0.753			
Social responsibility performance	SRP9	0.768	0.687	1.747	5.636	60.620
	SRP7	0.766	0.709			
	SRP8	0.740	0.677			
	SRP10	0.737	0.734			
	SRP6	0.723	0.664			
	SRP11	0.772	0.733			
	SRP12	0.731	0.682			
	SRP13	0.704	0.675			
Economic responsibility performance	EcRP15	0.776	0.717	1.573	5.074	65.694
	EcRP16	0.774	0.711			
	EcRP14	0.730	0.660			
	EcRP18	0.641	0.609			
	EcRP17	0.640	0.578			

**Table 7:** Model fitness and R^2^.

**Indices**	**One-Factor Model**	**Mediation Model**	**Recommended Level**
x2(DF)	100.324 (53)	804.383 (427)	
CMIN/DF (*p*-value)	1.893	1.884	<2.00
CFI	0.976	0.924	>0.90
TLI	0.970	0.917	>0.90
SRMR	0.025	0.052	<0.08
RMSEA	0.060	0.060	<0.07
**Indices**	**(R^2^)**	**(R^2^)**	
CSRP	24.6%	42.0%	
Economic		31.5%	
Environmental		26.4%	
Social		54.6%	

**Table 8:** Path coefficient and T statistics of direct effects.

Path	Original Sample	Sample Mean	Std. Beta	*T*-Statistics	*p*-Value
OSP-> CSR	0.091	0.090	0.068	1.343	0.180
OSP-> Economic	0.481	0.481	0.069	7.018	0.000
OSP-> Environment	0.458	0.457	0.061	7.497	0.000
OSP-> Social	0.686	0.680	0.056	12.323	0.000
Economic-> CSR	0.138	0.136	0.086	1.618	0.106
Environment-> CSR	0.321	0.320	0.062	5.222	0.000
Social-> CSR	0.217	0.225	0.083	2.633	0.009
